# Warfarin-Related Nephropathy Manifested as Diffuse Mesangial Proliferative Glomerulonephritis

**DOI:** 10.7759/cureus.22284

**Published:** 2022-02-16

**Authors:** Frederick Acquah, Nagapratap Ganta, Dina Alnabwani, Cecily Alaan, Priya Anantharaman, Pramil Cheriyath

**Affiliations:** 1 Internal Medicine, Hackensack Meridian Health, Ocean University Medical Center, Brick, USA; 2 Nephrology, Hackensack Meridian Health, Ocean University Medical Center, Brick, USA

**Keywords:** supratherapeutic inr, chronic kidney disease, acute kidney injury, diffuse mesangial proliferative glomerulonephritis, anticoagulant related nephropathy, warfarin related nephropathy

## Abstract

Warfarin is associated with anticoagulant-related nephropathy (ARN), one of the potential side effects. This is evidenced by a progressively increasing number of detected cases of deterioration in the kidney function even in patients with normal baseline function (GFR {glomerular filtration rate}) in addition to the chronic kidney disease (CKD) patients who are already vulnerable to ARN. There has been a clinical correlation in a rapid decline of kidney function and international normalized ratio (INR) levels of greater than three. ARN is a significant but underdiagnosed complication of anticoagulation that is associated with increased renal morbidity and all-cause mortality. We want to emphasize the importance of monitoring kidney function regularly and adjusting the appropriate doses of warfarin. We present a case of a 57-year-old female who was on warfarin for the mechanical aortic valve, presented with acute kidney injury and supratherapeutic INR. Her renal biopsy showed diffuse mesangial proliferative glomerulonephritis.

## Introduction

Warfarin-related nephropathy (WRN), also referred to as anticoagulant-related nephropathy (ARN) is a type of acute kidney injury (AKI) that is caused by excessive anticoagulation with warfarin and other anticoagulants [1]. Warfarin is the most appropriate anticoagulation regimen in those with prosthetic thrombosis, as most of the direct factor Xa inhibitors have not been studied enough for the prevention of thromboembolic events in patients with mechanical valve prosthesis, and excessive thrombotic complications owed to direct thrombin inhibitors [2]. It is very affordable, highly effective, for all these reasons. Warfarin is one of the most prescribed anticoagulants in the United States with an estimated number of prescriptions of 147,772,355 in 2018 [3]. Warfarin has been related to anticoagulant-related nephropathy (ARN) as one of the potential side effects, with progressively increasing in the number of detected cases of deterioration in the kidney function in patients with normal baseline function in addition to the chronic kidney disease (CKD) patients who are already vulnerable to ARN [2,4,5]. Lack of proactive measures in the diagnosis and timely intervention can result in patients requiring hemodialysis, especially ARN is more common in women and elderly populations [6]. Independent of the chronic kidney disease status, glomerular hemorrhage and renal tubular obstruction by red blood cells can be seen in patients who were treated with excessive warfarin. The underlying molecular mechanism for WRN was thought to be warfarin-induced thrombin depletion; a similar mechanism of action may also be seen with other anticoagulants such as dabigatran [7]. However, newer studies have hinted at an alternative mechanism involving reductions in activated protein C and endothelial protein C receptor signaling [8]. ARN, at least partially, is mediated via protease-activated receptor-1 (PAR-1). Mesangial proliferative glomerulonephritis (MPGN) is characterized by a diffuse or focal increase in the number of mesangial cells and expansion of the extracellular matrix in glomerular mesangium with or without immunoglobulin or complement deposition [9]. We report an interesting case of a 57-year-old female on warfarin for mechanical aortic valve who presented with AKI requiring hemodialysis and was found to have diffuse mesangial proliferative glomerulonephritis and warfarin-related nephropathy on renal biopsy.

## Case presentation

A 57-year-old Caucasian female presented to the emergency department with complaints of worsening fatigue and hematuria for two weeks. The patient states that she chronically takes over-the-counter Advil to help with mild headaches and occasional aches and pains. Her urine appeared light red to pink initially but progressed to becoming darker during the same period. She complained of dysuria, urgency, and increased frequency to her primary care physician (PCP) who prescribed nitrofurantoin after a urinalysis showed only 3+ occult blood and 2+ protein. The patient was unable to tolerate this antibiotic due to weakness, nausea, and vomiting, so she was transitioned to ciprofloxacin to begin a day before arrival to be taken for a week. Blood work obtained during the initial PCP visit was significant for an acute kidney injury with a creatinine of 3.1 mg/dL. After the results were discussed with her, it was recommended that she return to the office to repeat the basic metabolic panel (BMP). She denied chest pain, shortness of breath, palpitations, light-headedness, dizziness, vomiting, loss of appetite, diarrhea, or diaphoresis.

Past medical history of ischemic stroke with the left-sided deficit, stage 1 left breast cancer s/p lumpectomy and radiation, bicuspid aortic valve s/p valve replacement in 1996 on warfarin therapy, hypertension, hyperlipidemia, gastroesophageal reflux disease, and restless leg syndrome. Psychiatric history was pertinent for major depressive disorder and general anxiety disorder. Family history was non-contributory. Home medications included warfarin, aspirin, nifedipine, lisinopril, furosemide, potassium chloride tablets, lansoprazole, bupropion, fluoxetine, alprazolam, trazodone, and ropinirole. The patient was a current smoker with a 40-pack-year smoking history, occasional use of alcohol but denied use of illicit drugs. Review of systems (ROS) was positive for nausea, dysuria, flank pain, increased frequency, hematuria, weakness, and anxiety.

Vitals signs showed a temperature of 98.2°F, blood pressure of 130/61 mmHg, a pulse of 67 beats per minute, respiratory rate of 18 breaths per minute, pulse oximetry of 96% on room air. On physical examination, the patient had a dry mucous membrane, tongue deviated to right from midline with the strength of 3/5 in both left upper and lower extremities; a residual from prior CVA, audible wheezing in bilateral lung bases on auscultation and prosthetic heart valve sounds. Pertinent laboratory findings were shown in Table 1. The other labs such as serum electrolytes were normal except for slightly elevated potassium at 5.3 mmol/L (3.5-5.2 mmol/L) and liver function tests were normal. The patient's baseline labs were normal. Urinalysis showed large microscopic and macroscopic blood, WBC 60-80 cells/HPF (0-2 cells/HPF) with rare bacteria, and 2+ protein.

**Table 1:** Patient’s laboratory findings with reference range WBC: white blood cells, BUN: blood urea nitrogen, INR: international normalized ratio

Labs	Patient’s value	Reference range
Hemoglobin	10.8 g/dL	13.2-17.5 g/dL
WBC	7.1x10^3^/uL	4.5-11.0x10^3^/uL
Platelet count	274x10^3^/uL	140-450x10^3^/uL
INR	3.71	0.88-1.15
BUN	51 mg/dL	5-25 mg/dL
Serum creatinine	5.86 mg/dL	0.61-1.24 mg/dL

**Figure 1:**
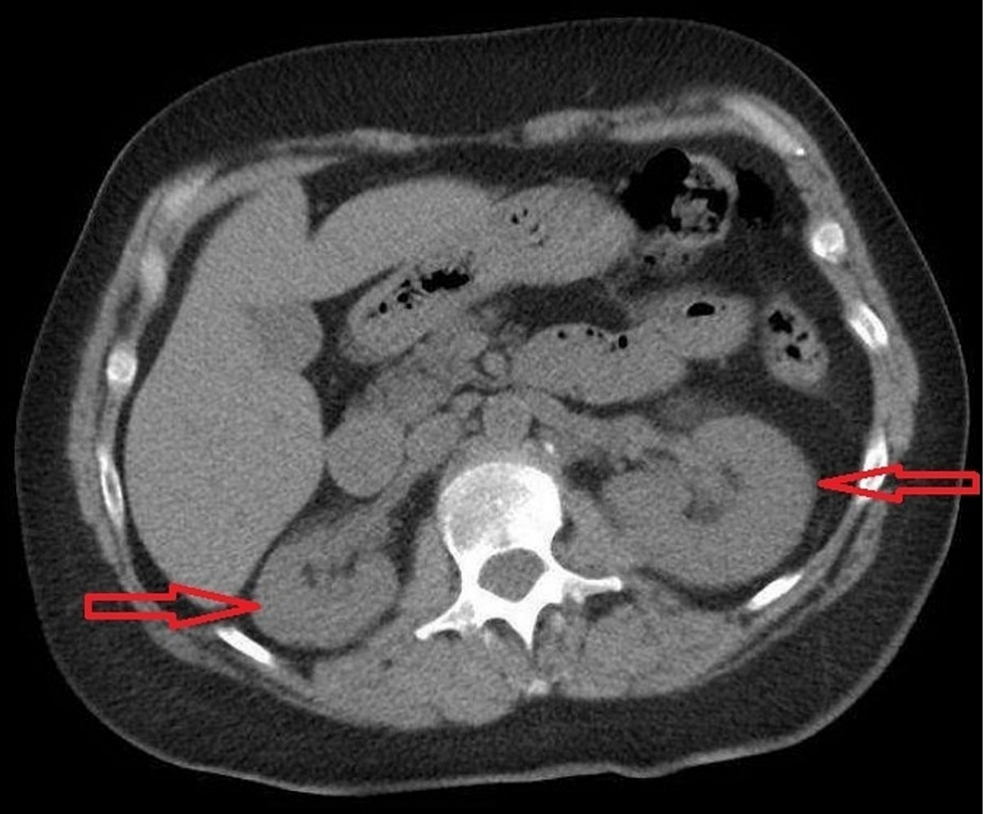
Computerized tomography (CT) abdomen and pelvis without contrast revealed no urolithiasis or hydronephrosis

EKG showed normal sinus rhythm with indeterminate axis low voltage QRS and right bundle branch block. Chest x-ray showed no acute pulmonary disease. CT abdomen pelvis without contrast revealed no urolithiasis or hydronephrosis but showed cholelithiasis (Figure 1).

Renal ultrasound findings were suggestive of intrinsic medical renal disease with no obstructive uropathy. Immunologic serologies such as antinuclear antibodies (ANA), antineutrophil cytoplasmic antibodies (ANCA), cryoglobulin, antistreptolysin O, anti-dsDNA, anti-glomerular basement membrane (anti-GBM) antibody, and C3 and C4 antibodies all came back as negative. The patient received pulse steroids but with worsening kidney functions, a vasc-cath was placed and hemodialysis was initiated. Renal biopsy was obtained six days after admission as the patient's international normalized ratio (INR) was not in the therapeutic range suitable to perform the procedure. Warfarin therapy was stopped to naturally correct the supratherapeutic INR due to the patient's metallic prosthetic aortic valve. Urology investigated the hematuria with a cystoscopy which was unremarkable. Kidney biopsy pathology under electron microscopy revealed diffuse mesangial proliferative glomerulonephritis with mild focal exudative features, immune complex type. Furthermore, it showed red blood cell casts, multifocal, with diffuse acute tubular injury consistent with warfarin nephropathy. The patient’s admission was further complicated by an unwitnessed mechanical fall leading to a left femoral fracture. She was status-post left hip hemiarthroplasty. The patient was discharged to subacute rehabilitation for reconditioning and to continue with outpatient hemodialysis for a few more months.

## Discussion

Warfarin-related nephropathy (WRN) is a common complication of warfarin therapy. However, it is underdiagnosed, as nephrologists are hesitant to perform a kidney biopsy on patients taking anticoagulants [10]. In a study involving 15,258 patients taking warfarin with an INR >3, 20.5% developed AKI within one week [11,12]. Risk factors that predispose one to have WRN are CKD, old age, diabetes mellitus, diabetic nephropathy, hypertension, and cardiovascular disease, specifically heart failure, and GN [10,12]. In the same study, 33.0% of those who have CKD had WRN as opposed to 16.5% of those who do not have CKD. They concluded that the one-year mortality for those with CKD irrespective of WRN was 31.1%. Non-CKD patients had one-year mortality of 18.9% [11].

WRN is an acute kidney injury without a known etiology in a supratherapeutic setting on an INR greater than 3.0 [8]. Brodsky et al. also classified WRN as renal biopsy findings of dysmorphic RBCs in the glomerulus, hemorrhage through all fields, and no active glomerulonephritis or inflammatory changes [11]. Presently, WRN is known as anticoagulant-related nephropathy (ARN) as it is also seen in other anticoagulants [12]. As in the patient above, renal biopsy showed diffuse mesangial proliferative glomerulonephritis with mild focal exudative features, immune complex type suggestive of resolving infection-related glomerulonephritis. Furthermore, it showed red blood cell casts, multifocal, with diffuse acute tubular injury consistent with warfarin nephropathy.

The nephropathy is caused by disruption of the glomerular filtration barrier provoking hemorrhage in the Bowman’s space and renal tubules, leading to the production of RBC casts causing obstruction, ischemia, and obliteration of the renal tubules [8,13]. The decrease in the kidney’s nephron mass due to the ischemia and the remaining tubules injured by the hyperfiltration will advance to CKD at an accelerated rate [8,14]. Patients may present with hypertension, volume overload, poor urine output, hematuria (gross or microscopic), proteinuria, and elevated creatinine. Prompt recognition of ARN is critical, as it is associated with accelerated progression of chronic kidney disease, and significant increases in short-term and long-term all-cause mortality.

Glassock has suggested the following criteria for the diagnosis of ARN: (1) history of hematuria, AKI, or worsening CKD; (2) usage of warfarin or novel oral anticoagulants before the onset of symptoms; (3) warfarin with INR above 3.0; (4) no acute hemorrhage and; (5) no other causes of AKI or hematuria should be suspected of WRN or ARN [12,15].

They should be worked up with urinalysis, urine electrolytes analysis, and kidney ultrasound [8]. Although hematuria is a common finding in WRN, its absence does not rule it out. Therefore, any patient who presents with an AKI with a history of supratherapeutic INR and no known etiology should be considered as WRN. Treatment for WRN/ARN is supportive. The anticoagulant should be adjusted within the therapeutic range [8].

Wheeler et al. made the following recommendations for the prevention of WRN and monitoring those who use warfarin: (1) check INR and kidney function every three to four weeks during the first three months of anticoagulation, (2) monitor the renal function every three to six months with those who have a creatinine clearance of <60 mL/min, (3) assess the renal function of any patient with supratherapeutic INR as soon as possible, (4) do renal workup on any patient on anticoagulants with acute worsening of renal function [8].

Our patient’s INR level from the past five years has fluctuated with it being mostly supratherapeutic (range: 1.13-7.37). However, her serum creatinine was within normal limits (<1.0 mg/dL) until recently when she developed a UTI and it was found to be 3.1 mg/dL. Although she was treated with antibiotics and steroids her kidney function continued to worsen leading to her need for dialysis. Based on her kidney biopsy, her diffuse MPGN which was superimposed with WRN could have been attributed to her acute renal failure. Unless her INR is returned within the therapeutic range, she will continue to have WRN which would further damage her kidneys. Although WRN is common among patients taking warfarin, it is underdiagnosed leading to delay of proper treatment which can cause further complications or damage to the kidneys.

## Conclusions

Warfarin-related nephropathy or anticoagulant-related nephropathy should be considered if severe warfarin coagulopathy is present and if other causes of acute kidney injury have been excluded in patients on chronic anticoagulant therapy. Coagulopathy, especially if INR is greater than four, CKD is the strongest risk factor for the development of warfarin-related nephropathy. Renal function should be regularly monitored in patients on anticoagulant therapy. Any patient receiving anticoagulation with acute worsening of renal function needs an immediate renal workup, including urine analysis, urine electrolyte analysis, and renal ultrasound. If that workup is negative or demonstrates isolated hematuria, ARN should be strongly considered in the differential diagnosis. ARN can be prevented by adjusting warfarin dosage especially in patients with CKD. Recovery of renal function in patients with anticoagulant-related nephropathy varies, with some patients reverting back to baseline once INR is stabilized while some patients may not have any recovery. The role of steroids in warfarin-related nephropathy is unclear which should be investigated in further studies.
